# Proton beam therapy for the isolated recurrence of endometrial cancer in para-aortic lymph nodes: a case report

**DOI:** 10.1186/s12905-022-01961-1

**Published:** 2022-09-14

**Authors:** Kaname Uno, Masato Yoshihara, Sho Tano, Takehiko Takeda, Yasuyuki Kishigami, Hidenori Oguchi

**Affiliations:** 1 Department of Obstetrics and Gynecology, TOYOTA Memorial Hospital, Toyota, Aichi Japan; 2 Department of Obstetrics and Gynecology, Nagoya University Graduate School of Medicine, Nagoya, Aichi Japan; 3 Division of Clinical Genetics, Department of Laboratory Medicine, Lund University Graduate School of Medicine, Lund, Sweden

**Keywords:** Endometrial cancer, Proton beam therapy, Recurrence, Para-aortic lymph node, Case report

## Abstract

**Background:**

Proton beam therapy penetrates tumor tissues with a highly concentrated dose. It is useful when normal structures are too proximate to the treatment target and, thus, may be damaged by surgery or conventional photon beam therapy. However, proton beam therapy has only been used to treat recurrent endometrial cancer in a few cases; therefore, its effectiveness remains unclear.

**Case presentation:**

We herein report a case of the isolated recurrence of endometrial cancer in the para-aortic lymph nodes in a 59-year-old postmenopausal woman that was completely eradicated by proton beam therapy. The patient was diagnosed with stage IIIC2 endometrial cancer and treated with 6 courses of doxorubicin (45 mg/m^2^) and cisplatin (50 mg/m^2^) in adjuvant chemotherapy. Fifteen months after the initial therapy, the isolated recurrence of endometrial cancer was detected in the para-aortic lymph nodes. The site of recurrence was just under the left renal artery. Due to the potential risks associated with left kidney resection due to the limited surgical space between the tumor and left renal artery, proton beam therapy was administered instead of surgery or conventional photon beam therapy. Following proton beam therapy, the complete resolution of the recurrent lesion was confirmed. No serious complications occurred during or after treatment. There have been no signs of recurrence more than 7 years after treatment.

**Conclusions:**

Proton beam therapy is a potentially effective modality for the treatment of recurrent endometrial cancer where the tumor site limits surgical interventions and the use of conventional photon beam therapy.

## Background

Proton beam therapy (PBT) is one of the most effective and widely used particle therapies because of its ability to penetrate tumor tissues with a highly concentrated dose [1]. The superiority of PBT is particularly useful when normal structures are too proximate to the treatment target and, thus, may be damaged by surgery or conventional photon beam therapy [2, 3]. Although the effectiveness of PBT for cervical cancer has been demonstrated in clinical studies [4, 5], its utility for the treatment of endometrial cancer remains unclear [6]. We herein report a case of the isolated recurrence of endometrial cancer in the para-aortic lymph nodes that was completely eradicated with PBT.

## Case presentation

**Fig. 1:**
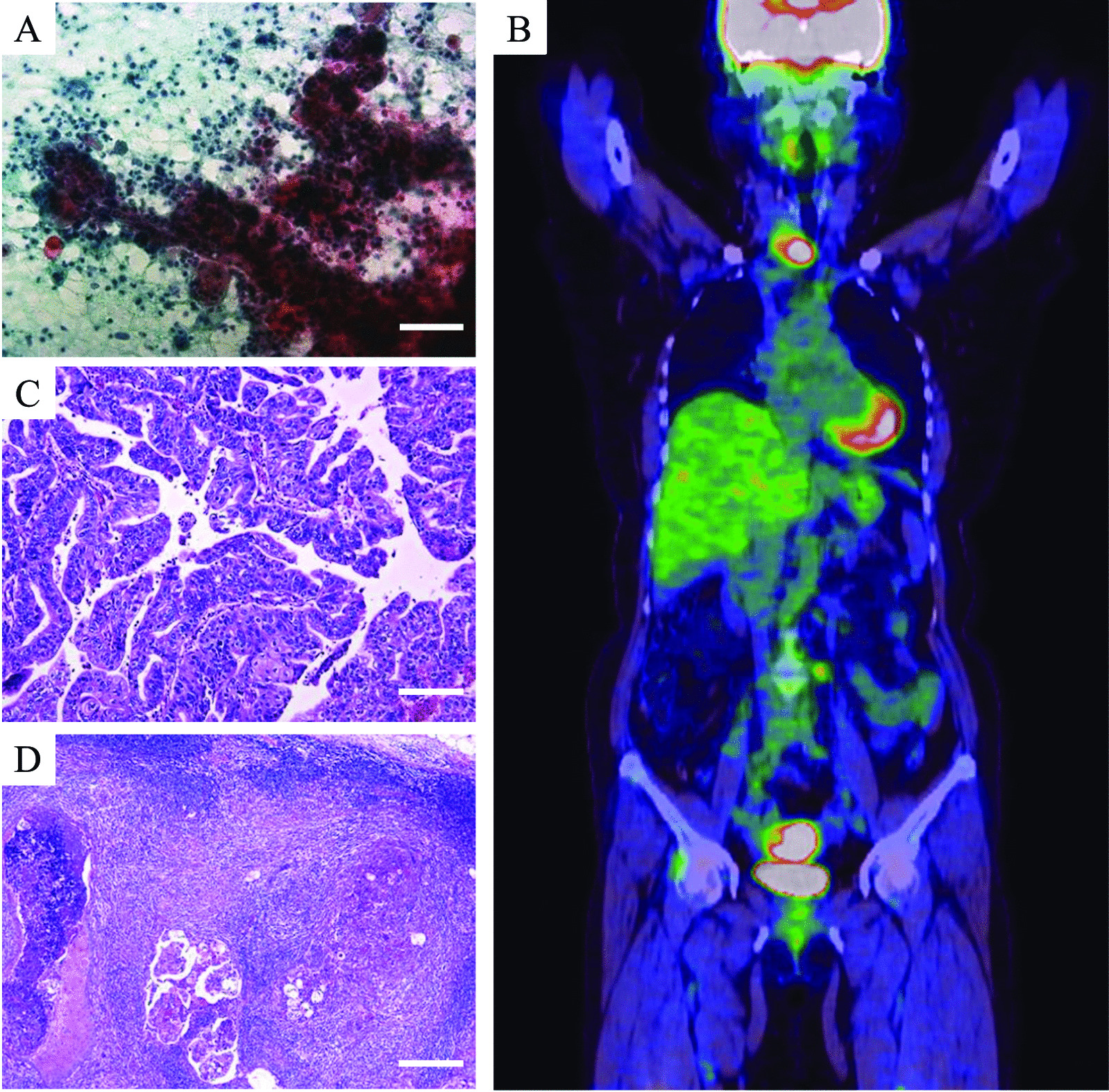
An endometrial Pap smear strongly indicated adenocarcinoma (at 20 × magnification, scale bar: 100 μm) (**A**). Positron emission tomography/computed tomography detected the abnormal accumulation of 2-deoxy-2-(18F)fluoro-d-glucose in the uterine corpus (SUVmax = 16.9) and para-aortic lymph nodes (SUVmax = 4.1) (**B**). The histopathological finding of endometrial carcinoma was compatible with grade 1 adenocarcinoma of the uterus (at 20 × magnification, scale bar: 100 μm) (**C**) and metastasis to the para-aortic lymph nodes (at 4 × magnification, scale bar: 500 μm) (**D**)

A 59-year-old postmenopausal woman (gravida 3, para 3) was referred to our hospital with an abnormal endometrial cytology and thickening. Her medical history included obesity and partial thyroidectomy for thyroid cancer at the age of 49 years. Her social and family histories were unremarkable. An internal examination revealed that the uterus was the size of a goose egg, transvaginal ultrasonography showed an enlarged uterus with thickening of the endometrium to 19.5 mm, and deep myometrial invasion was suspected. Laboratory results showed elevated serum cancer antigen (CA) 125 and CA19-9 levels (129 and 117 U/mL, respectively). An endometrial Pap smear revealed suspected adenocarcinoma (Fig. 1a), while biopsy results confirmed the existence of endometrioid adenocarcinoma. Magnetic resonance imaging of the abdomen revealed a local, space-occupying lesion arising from the anterior uterine wall, while positron emission tomography/computed tomography (PET/CT) showed the abnormal accumulation of 2-deoxy-2-(18F)fluoro-d-glucose (FDG) in the uterine corpus with a maximum standardized uptake value (SUVmax) of 16.9 as well as in the bilateral iliac and para-aortic lymph nodes (SUVmax = 4.1) (Fig. 1b).

The patient underwent extended hysterectomy, bilateral salpingo-oophorectomy, omentectomy, and pelvic and para-aortic lymphadenectomy below the renal veins. A histopathological examination revealed grade 1 endometrioid carcinoma of the uterine corpus (Fig. 1c), with myometrial invasion of > 50% and metastases of the bilateral iliac and para-aortic lymph nodes (Fig. 1d). According to the International Federation of Gynecologists and Obstetricians guidelines, the lesion was diagnosed as stage IIIC2 endometrial cancer. After surgery, adjuvant therapy with six courses of doxorubicin (45 mg/m^2^) and cisplatin (50 mg/m^2^) successfully inhibited disease progression.

**Fig. 2:**
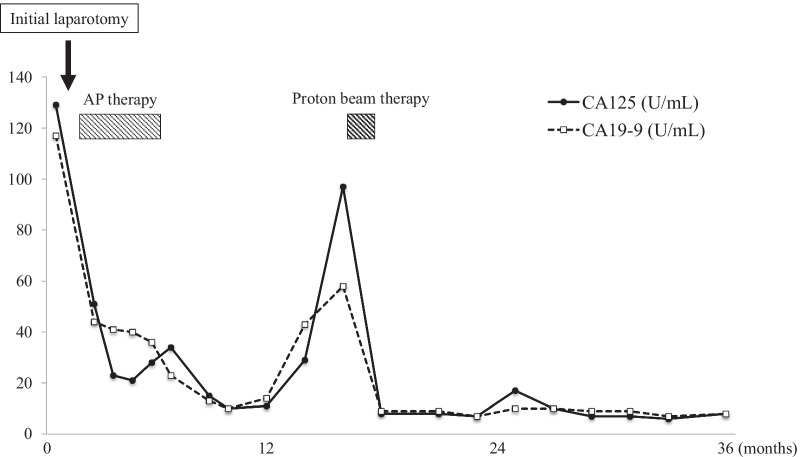
The 3-year clinical course of the patient from the initial diagnosis is highlighted by serum levels of cancer antigen (CA) 125 and CA19-9. AP, doxorubicin, and cisplatin therapy

**Fig. 3:**
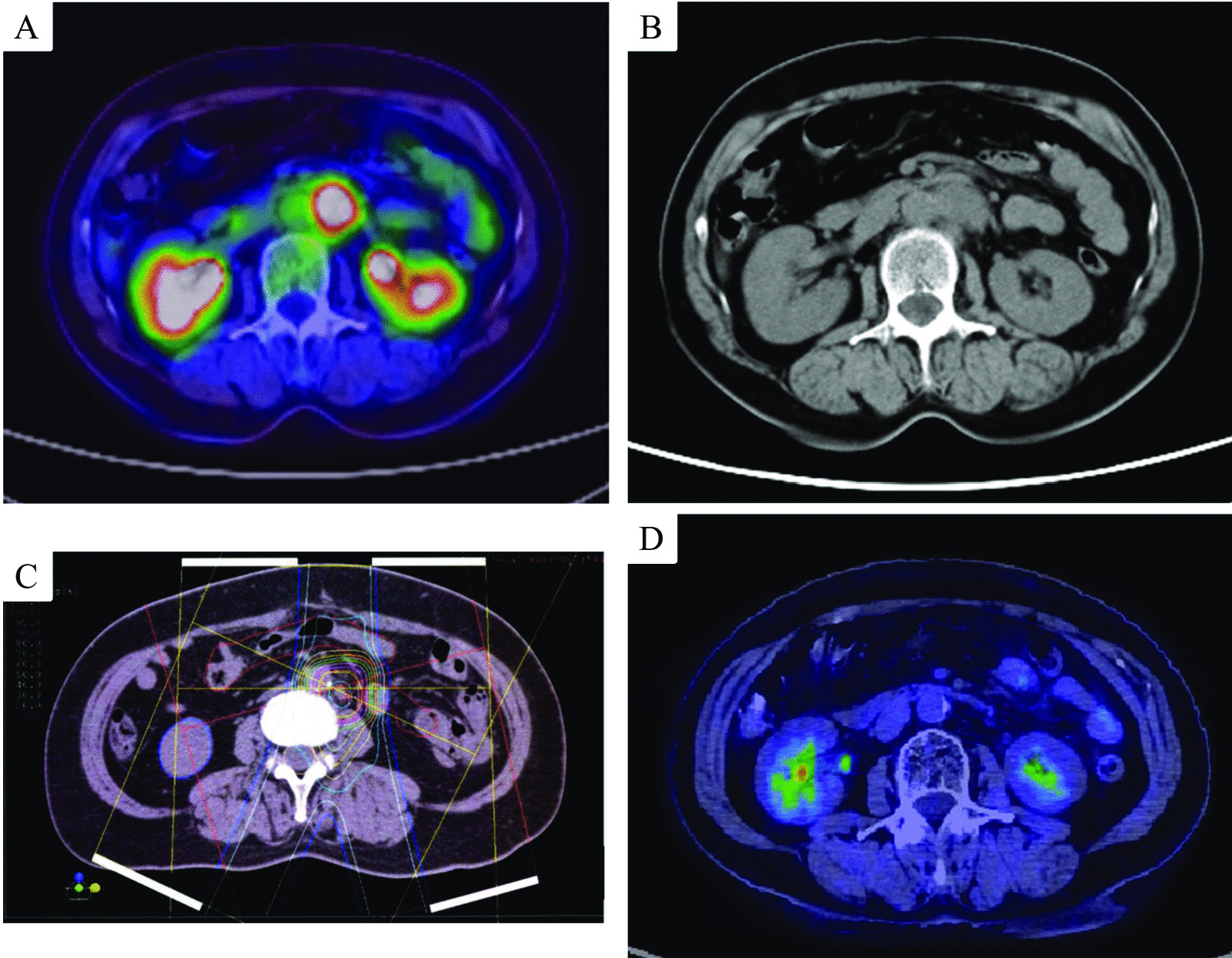
Positron emission tomography/computed tomography (PET/CT) scans before proton beam therapy (PBT) (**A**). A solid tumor with the abnormal accumulation of 2-deoxy-2-(18F)fluoro-d-glucose (SUVmax = 19.44) was observed adjacent to the left renal vein (**B**). A dose distribution curve of the axial and sagittal sections **C** orange, purple, pink, green, yellow, sky blue, and dark blue lines in the circle represent doses of 5225, 4950, 4400, 3850, 2250, 1650, and 550 cGyE, respectively, which were delivered to the patient over the course of the PBT regimen. PET/CT scans of the para-aortic lesion after PBT (**D**). The recurrent para-aortic lesion was completely eradicated

The clinical course of the patient is shown in Fig. 2. Elevated levels of CA125 and CA19-9 gradually decreased and normalized after surgery and adjuvant chemotherapy. However, fifteen months after the initial surgery, serum CA125 and CA19-9 levels spontaneously increased, and the recurrence of endometrial cancer in the para-aortic lymph nodes was confirmed by the abnormal accumulation of FDG (SUVmax = 19.44) on a PET/CT scan (Fig. 2, 3a, b). The recurrent site in the para-aortic lymph nodes was located just under the left renal artery. Due to the potential influence of the proximal left kidney to limit the surgical resection of the tumor, the patient chose to undergo PBT at an advanced medical center that specializes in radiology. Thirty-six days after the diagnosis of recurrence, PBT was performed at a total dose of 68.2 GyE at 2.2 GyE per fraction (Fig. 3c) for 51 days. No serious complications were observed during PBT. Serum CA125 and CA19-9 levels immediately decreased to within normal ranges of 8 and 9 U/mL, respectively, after PBT. The complete resolution of the recurrent lesion was confirmed in follow-up PET/CT (Fig. 3d). The patient did not receive any additional treatment after PBT. Complications related to PBT, including dermatitis, nausea, anemia, and leukopenia, did not occur after treatment. Careful follow-ups after PBT were performed every three months and involved a physical examination, transvaginal ultrasound, and laboratory tests (including CA125 and CA19-9). Follow-up CT was performed at least every 6 months. CA125 and CA19-9 levels have remained stable within normal ranges after treatment. The condition of the patient is good 7 years after PBT with no signs of recurrence.

All cytology and pathological images were obtained with an Olympus BX51 microscope (Olympus, Tokyo, Japan) with 4 × , 10 × , 20 × , 40 × UPlanSApo objective lenses (Olympus), which equipped with a DP27 digital camera (Olympus) and model U-TV0.5XC-3 TV system (Olympus) on upper site of the microscope. These images were captured by the digital ruler of image analysis software cellSens standard version 1.16 (Olympus). All images were adjusted automatically about white balance and resolution in the software.

## Discussion and conclusions

Protons are widely recognized to possess physical and biological properties that are superior to photons for conventional use [1, 3]. Protons are large positively-charged particles that penetrate tissues to various depths and deposit most of their energy in targeted tissues, which is referred to as the Bragg peak [7]. The physical properties of protons may maximize tumor-focused radiation and minimize effects on the surrounding tissues. In addition, the relative biological effectiveness of protons reaches 1.1 or potentially higher in the distal part of the spread-out Bragg peak [1]. Despite the theoretical concerns of damage to adjacent normal tissues associated with its greater biological effect, a large retrospective study repudiated the higher risk of secondary malignancies in patients who have undergone PBT than in those who have undergone photon therapy [8]. Although its use in clinical applications remains challenging because of the equipment required and associated costs, PBT provides an ideal radiation regimen based on its physical and biological features.

Since 1954, when the medical use of protons was initially reported, PBT has been applied to various parts of the body, including the uvea, skull base, and spine [1, 9, 10]. In the gynecological field, the clinical use and efficacy of PBT have mainly been reported for cervical carcinoma [3–5], low-grade endometrial stromal sarcoma [11], and solitary recurrent epithelial ovarian cancer [12]. Only one case report of the vaginal recurrence of endometrial carcinoma treated with PBT is available in the literature, which describes the complete regression of the tumor; however, there was a complication of grade 1 cystitis. This case report indicated the potential efficacy of PBT for recurrent endometrial cancer [6]. Unlike vaginal recurrence, curative radiation therapy is not widely recommended for the recurrence of endometrial cancer in the para-aortic lymph nodes. The number of patients with endometrial cancer has recently been increasing worldwide [13]. Recurrence has been detected in approximately 20% of patients with endometrial cancer [14]. Therefore, the number of patients with recurrent endometrial cancer has also been increasing [13]. Recurrent endometrial cancer often occurs in lymph nodes [14, 15]. Therefore, the careful consideration of treatment strategies for recurrent endometrial cancer in para-aortic lymph nodes is essential to improve the prognosis and quality of life of patients during the treatment. In the present case, PBT effectively eradicated the isolated recurrence of endometrial cancer in the para-aortic lymph nodes adjacent to the left renal vein, the location of which impeded surgical interventions. Due to the limitations associated with conventional photon therapy, PBT was considered to be the preferred choice for our patient at that time.

No case reports in gynecological cancer have reported severe complications related to PBT. Previous studies reported transient low-grade cystitis or fever only as complications [6, 12]. A randomized phase IIB trial of esophageal cancer revealed that PBT was associated with fewer complications than intensity-modulated radiation therapy using conventional photons [16]. A systematic review of esophageal cancer showed that PBT has the potential to not only improve patient outcomes, but also reduce complications [17]. Since PBT maximizes tumor-focused radiation and minimizes effects in the surrounding tissues, there are very few case reports of complications related to the suppression of bone marrow [18]. In the present case, there were no signs of the suppression of bone marrow during or after PBT. Therefore, PBT is associated with fewer complications than conventional photon treatments, which is beneficial.

To the best of our knowledge, this is the first case report to describe the successful treatment of the isolated distant recurrence of endometrial cancer by PBT, which may be regarded as a potentially effective treatment modality for recurrent endometrial cancer where the tumor location limits the application of surgery or conventional photon beam therapy. PBT is associated with fewer complications than conventional radiotherapy. The further accumulation of cases and additional trials are needed to establish the effectiveness of PBT for recurrent endometrial cancer as well as uterine cervical cancer.

## Data Availability

Not applicable.

## Abbreviations

PBT: Proton beam therapy
CA: Cancer antigen
PET/CT: Positron emission tomography/computed tomography
FDG: Fluoro-d-glucose
SUV: Standardized uptake value
